# Supplementary material for: A Genome-Scale Metabolic Model of Thalassiosira pseudonana CCMP 1335 for a Systems-Level Understanding of Its Metabolism and Biotechnological Potential
Source: Microorganisms. 2020 Sep 11;8(9):1396. doi: 10.3390/microorganisms8091396 (PMC7563145; doi:10.3390/microorganisms8091396)
Supplement: Supplementary file 1 [file microorganisms-08-01396-s001.zip › S07_Methods_Growth.docx]

**Methods**

**Culture conditions**

*Thalassiosira pseudonana* was procured from CCAP culture collection facility in UK. The strain was cultured in f/2 medium + Si^+^ [1] at 18-20 °C under 16:8 light:dark hours with 150 μmol∙m^-2^∙s^-1^ of illumination in the light phase. The growth was measured by measuring the optical density at 600 nm (OD_600_) every 24 h for 8 days using a spectrophotometer.

**Estimation of Biomass Composition**

The biomass equation was formulated by experimental measurement of the major biomass components such as proteins, carbohydrates, lipids, DNA and RNA while the minor components, e.g., pigments and chitin, were taken from previously-published models[2].

The carbohydrate content was measured by the phenol-sulphuric acid method [3] with standard curve of known concentrations of glucose. The total protein was extracted in 1N NaOH [4]. The concentration of extracted proteins was measured by the bicinchoninic acid assay (BCA) method [5] (Pierce BCA Protein Assay Kit, Thermo Scientific, Rockford, IL) using Bovine serum albumin (BSA) (Sigma-Aldrich) as standard. Furthermore, the dried cells were hydrolysed with 6N HCl for 24 h at 105 ºC and subjected to amino acids derivatization to obtain distribution of individual amino acids using Gas Chromatography Mass Spectrometry (GSMS). After the derivatization, the amino acid samples were analyzed by GCMS with Agilent 7890A GC system having HP5 column (30m × 0.25mm ID, 0.25  thickness, Varian) coupled with Agilent 7000 QQQ MS. 1 ml of sample was injected in the split mode 10: 1. The signals were recorded in full scan mode (m/z 50 to 650, 250 scan/ms). The ionization energy was fixed to 70eV and 99.99% pure He was used as carrier gas with at the flow rate of 1.1 ml/min. The temperatures of mass transfer line and injector were set at 220°C and 250°C, respectively. The temperature of oven was programmed as follows: initially at 100 °C for 2 min, followed by increase by 5 °C/min up to 180°C and further increased by 10 °C/min up to 300 °C, hold at 300 °C for 0.5 min. Finally, the detected amino acid fragments and their respective retention times were recorded and identified by comparing the fragmentation pattern with NIST mass spectral library using AMDIS and mass hunter software. The concentrations of the amino acids not detected by this method were taken from the *P. tricornutum* model [2].

The total lipids were extracted by the Hexane-Isopropanol method [4] and measured gravimetrically. The extracted lipid was transesterified to form Fatty Acid Methyl Ester (FAME) and subjected to FAME analysis by GC-MS. The fatty acid composition of different lipids classes was obtained from previous study [6].

The quantity of DNA was measured using Hoechst 33258 dye [7]. The DNA was extracted in a rehydration buffer (0.5 g lysozyme in 50 mL Tris-EDTA buffer of pH = 8.0). One mL of the rehydration buffer was added to 10 mg of dried cells followed by 1h incubation at 37 ºC. A commercially available DNA powder (Salmon sperm DNA, Sigma-Aldrich) was used to prepare DNA standard solutions. The DNA standard solution and samples were processed similarly and the concentration of DNA was measured using Hoechst 33258 dye and measuring the fluorescence. RNA was extracted using KOH and perchloric acid method [8] and estimated spectrophotometrically by taking absorbance measurements at the 260 nm and 280 nm. The minor biomass component (pigments) was adopted from previous reports [2,6]. Chitin was also included in the biomass equation as *T. pseudonana* cell wall has chitin. The chitin content was not available so it was assumed to be 2% of total biomass.

**Results**

**Growth Profile**

The photoautotrophic growth of *T. pseudonana* was observed for eight days. The specific growth rate was found to be 0.024 h^-1^. The final OD (optical density) at the end of 8 days was found to be around 0.6.

**Figure S1**. Growth profile of T.pseudonana under photoautotrophic condition

O_2_ evolution Vs O_2_ Consumption


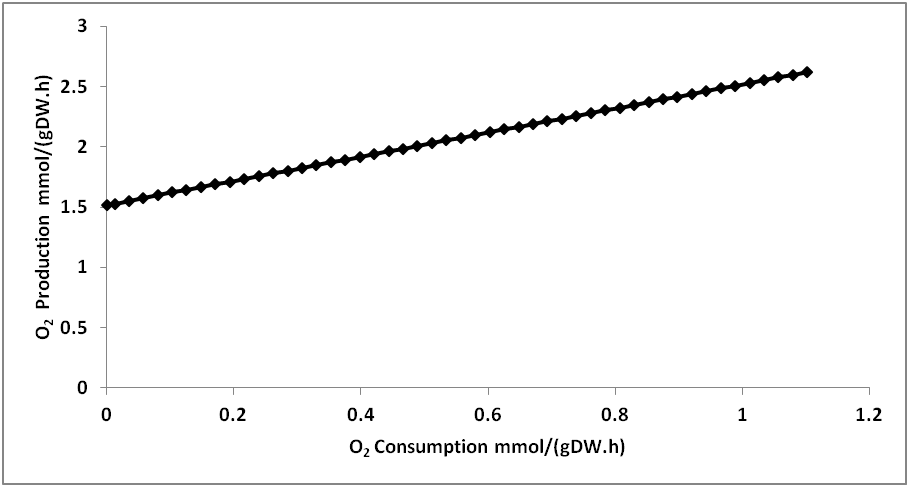


**Figure S2**: O_2_ consumption and O_2_ production are linearly correlated with increasing ATPM values.

Comparison of iThaps987 model with P. tricornutum model, iLB1025


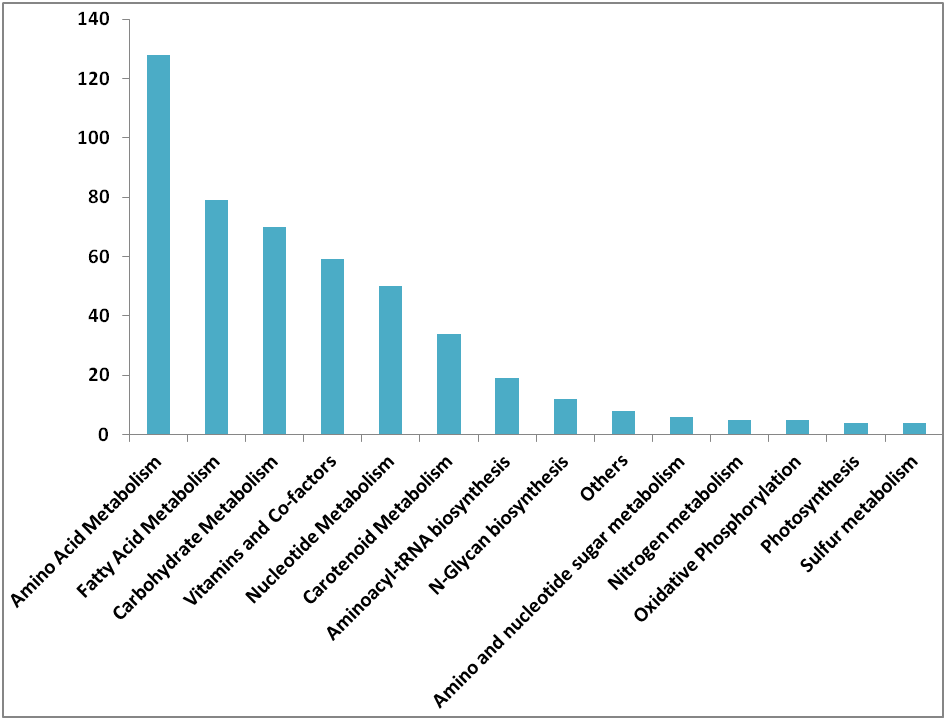


Figure S3: Distribution of common enzymes (between *iThaps987* and *iLB1025*) across different pathways.

References

1. Allen, A.E.; Dupont, C.L.; Oborník, M.; Horák, A.; Nunes-Nesi, A.; McCrow, J.P.; Zheng, H.; Johnson, D.A.; Hu, H.; Fernie, A.R.; et al. Evolution and metabolic significance of the urea cycle in photosynthetic diatoms. *Nature* **2011**, *473*, 203–207.

2. Levering, J.; Broddrick, J.; Dupont, C.L.; Peers, G.; Beeri, K.; Mayers, J.; Gallina, A.A.; Allen, A.E.; Palsson, B.O.; Zengler, K. Genome-Scale Model Reveals Metabolic Basis of Biomass Partitioning in a Model Diatom. *PLoS One* **2016**, *11*, e0155038.

3. DuBois, M.; Gilles, K.A.; Hamilton, J.K.; Rebers, P.A.; Smith, F. Colorimetric Method for Determination of Sugars and Related Substances. *Anal. Chem.* **1956**, *28*, 350–356.

4. Watson, J.; Degnan, B.; Degnan, S.; Krömer, J.O. Determining the Biomass Composition of a Sponge Holobiont for Flux Analysis. In; Humana Press, New York, NY, 2014; pp. 107–125.

5. Smith, P.K.; Krohn, R.I.; Hermanson, G.T.; Mallia, A.K.; Gartner, F.H.; Provenzano, M.D.; Fujimoto, E.K.; Goeke, N.M.; Olson, B.J.; Klenk, D.C. Measurement of protein using bicinchoninic acid. *Anal. Biochem.* **1985**, *150*, 76–85.

6. Yongmanitchai, W.; Ward, O.P. Separation of lipid classes from Phaeodactylum tricornutum using silica cartridges. *Phytochemistry* **1992**, *31*, 3405–3408.

7. Cesarone, C.F.; Bolognesi, C.; Santi, L. Improved microfluorometric DNA determination in biological material using 33258 Hoechst. *Anal. Biochem.* **1979**, *100*, 188–197.

8. Fleck, A.; Munro, H.N. The precision of ultraviolet absorption measurements in the Schmidt-Thannhauser procedure for nucleic acid estimation. *BBA - Biochim. Biophys. Acta* **1962**, *55*, 571–583.
